# Accuracy of ctDNA-based minimal residual disease detection in predicting postoperative recurrence of breast cancer: a meta-analysis

**DOI:** 10.3389/fonc.2026.1735752

**Published:** 2026-02-03

**Authors:** Hang You, JiuJiang He, Tian Tian

**Affiliations:** Department of Breast Surgery, The Affiliated Yongchuan Hospital of Chongqing Medical University, Chongqing, China

**Keywords:** breast cancer, circulating tumor DNA, ctDNA, diagnostic accuracy, monitoring strategy, postoperative recurrence

## Abstract

**Background:**

Detection of circulating tumor DNA (ctDNA) has attracted growing attention for predicting postoperative breast cancer recurrence; however, the differences between the landmark and surveillance strategies remain unclear.

**Methods:**

We systematically searched the PubMed, Cochrane Library, Embase, and Ovid MEDLINE databases for studies published up to April 17, 2025. Effect models were selected based on heterogeneity tests to pool diagnostic indicators, including sensitivity and specificity. Subgroup analyses were conducted according to molecular subtype, detection method, analytical strategy, and disease stage.

**Results:**

A total of 17 studies were included in the analysis. The sensitivity and specificity of the landmark strategy were 0.40 (95% CI: 0.22–0.62) and 0.95 (95% CI: 0.81–0.99), respectively. For the surveillance strategy, sensitivity was 0.79 (95% CI: 0.71–0.85) and specificity was 0.98 (95% CI: 0.92–0.99). The surveillance strategy significantly improved sensitivity without a substantial loss of specificity. Among molecular subtypes, triple-negative breast cancer(TNBC) exhibited the best performance under the surveillance strategy. Whole-genome sequencing (WGS), droplet digital PCR (ddPCR), and whole-exome sequencing (WES) all demonstrated high sensitivity within the surveillance framework.

**Conclusion:**

ctDNA serves as a highly specific biomarker for predicting postoperative breast cancer recurrence. The surveillance strategy substantially improves its sensitivity; however, the current performance remains below the ideal threshold for clinical implementation. Future research should focus on refining detection strategies and technologies to achieve personalized recurrence risk stratification and guide therapeutic decision-making.

**Systematic Review Registration:**

https://www.crd.york.ac.uk/prospero/, identifier CRD420251056270.

## Introduction

Breast cancer is now the most prevalent malignancy among women worldwide, accounting for approximately 25% of all female cancers, and its incidence continues to increase (1). Despite significant advances in diagnostic and detection technologies, breast cancer remains a leading cause of cancer-related mortality among women (2). According to the expression levels of progesterone receptor (PR), estrogen receptor (ER), and human epidermal growth factor receptor 2 (HER2), breast cancer is classified into luminal A, luminal B, HER2+, and triple-negative subtypes. Further refinements to this classification have been proposed as research progresses (3). Comprehensive treatment strategies for breast cancer, including surgery, chemotherapy, targeted therapy, endocrine therapy, and radiotherapy, have been continuously refined. Although more patients can now achieve remission or cure, surgery remains the cornerstone of curative treatment (4).

After curative treatment, conventional follow-up monitoring primarily depends on periodic imaging examinations, which can only detect recurrence once it becomes radiographically or clinically apparent. In recent years, liquid biopsy technologies have attracted increasing attention. ctDNA has emerged as a sensitive and specific biomarker for monitoring recurrence across multiple cancers, including breast cancer, thereby enabling early detection and informing adjuvant or consolidation therapy (5).

Current studies on ctDNA-based prediction of breast cancer recurrence mainly employ two analytical approaches: the landmark strategy and the surveillance strategy. The landmark strategy involves a single ctDNA measurement at a specific time point to predict recurrence risk, whereas the surveillance strategy entails longitudinal monitoring at multiple follow-up time points to assess treatment response and predict recurrence based on dynamic ctDNA fluctuations (6). Two major technical approaches have been established for ctDNA detection (1): the tumor-informed strategy, which involves sequencing tumor tissue to identify patient-specific mutations followed by targeted ctDNA tracking; and (2) the tumor-uninformed strategy, which employs predefined genomic panels to directly detect common cancer-related genetic or epigenetic alterations in plasma without prior tissue sequencing (7).

To evaluate the diagnostic accuracy of ctDNA in predicting postoperative breast cancer recurrence and to compare its performance across different detection strategies, we conducted a systematic meta-analysis of published studies. This analysis aimed to determine the pooled sensitivity and specificity of ctDNA as a prognostic biomarker and to perform subgroup analyses according to molecular subtype, tumor stage, and detection method.

## Methods

### Design

This systematic review and meta-analysis was conducted in accordance with the PRISMA reporting guidelines. The study protocol was prospectively registered in the PROSPERO International Register of Systematic Reviews (registration number: CRD420251056270).

### Literature search strategy

We searched the PubMed, Cochrane Library, Embase, and Ovid MEDLINE databases using three main topics—breast cancer, circulating tumor DNA (ctDNA), and minimal residual disease (MRD). Corresponding Medical Subject Headings (MeSH) and free-text terms were combined, restricting the search to studies published between 2000 and 2025. The final search was completed on April 17, 2025 (**Supplementary Table S1).**

### Study and patient selection

The inclusion criteria for this meta-analysis were as follows: (1) randomized controlled trials or prospective/retrospective cohort studies; (2) patients diagnosed with stage I–III breast cancer who underwent surgical treatment; and (3) studies reporting at least one postoperative ctDNA test result with corresponding recurrence outcomes. The exclusion criteria were: (1) sample size ≤10; (2) non-English publications or unavailable full text; and (3) absence of ctDNA or recurrence data.

### Data extraction and quality assessment

The extracted data included the first author, publication year, clinicopathological characteristics, detection methods, the number of ctDNA-positive and ctDNA-negative patients, and recurrence status. The sensitivity and specificity of ctDNA detection were calculated based on true positive (TP), false positive (FP), true negative (TN), and false negative (FN) results. The methodological quality of each study was evaluated using the Quality Assessment of Diagnostic Accuracy Studies-2 (QUADAS-2) tool (8), and the risk of bias was determined according to predefined criteria (**Supplementary Figure S1**).

### Statistical analysis

We used R software (version 4.5.1) and the meta package to estimate and evaluate diagnostic performance indicators, including sensitivity, specificity, positive likelihood ratio (PLR), negative likelihood ratio (NLR), and diagnostic odds ratio (DOR). In general, a PLR greater than 5.0 and an NLR less than 0.2 were considered clinically meaningful. Significant heterogeneity was considered present when P < 0.05 or I² > 50%, in which case a random-effects model was applied; otherwise, a fixed-effects model was used. Subgroup analyses were performed based on molecular subtype (TNBC, HER2+, HR+ HER2-), tumor stage (stage III ≥ 25.5% vs. < 25.5%), ctDNA detection method (WGS, WES, ddPCR, next-generation sequencing [NGS]), and detection strategy (tumor-informed vs. tumor-uninformed). The proportion of stage III breast cancer patients was calculated for studies reporting detailed staging data. These studies were then categorized into two groups according to the median proportion of stage III cases (**Supplementary Table S2**).

## Results

### Systematic review and study characteristics

**Figure 1:**
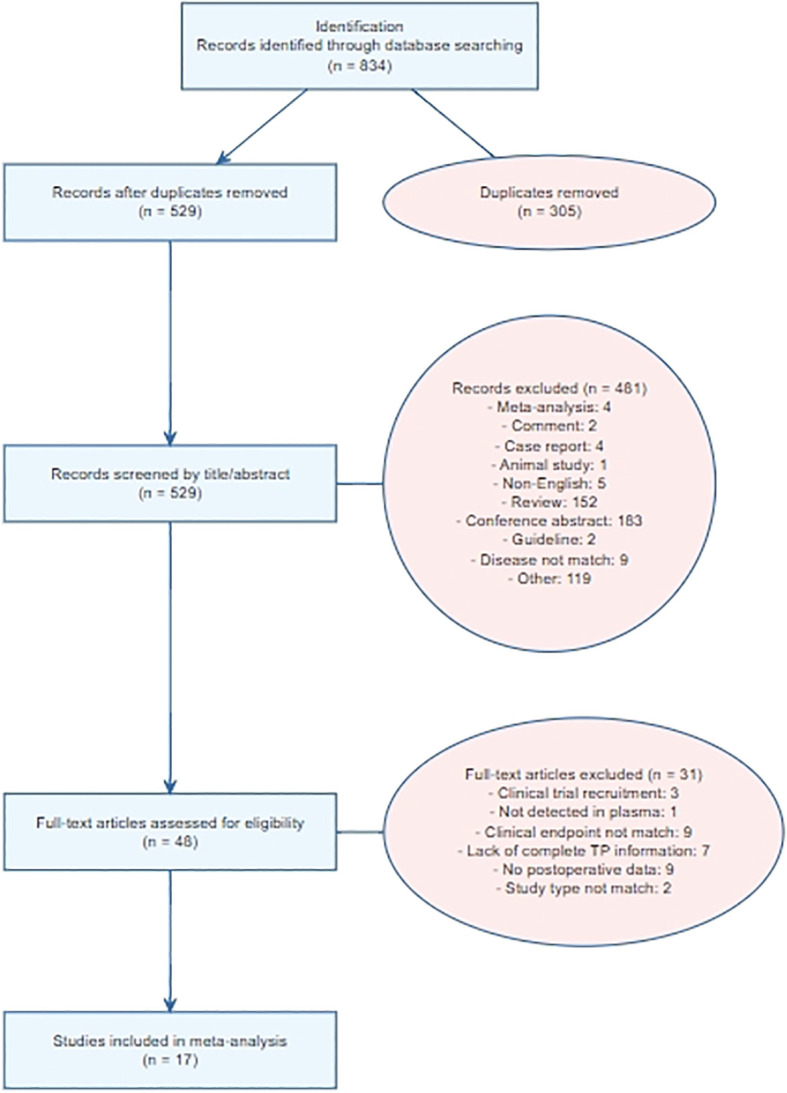
Flow diagram for selection of studies.

A total of 834 articles were identified through database searches, and 17 studies were ultimately included in the final analysis (5, 9–24) (**Figure 1**). The analysis included 698 patients from 11 studies using the landmark strategy and 735 patients from 11 studies using the surveillance strategy. Among these, seven studies specifically investigated TNBC, one study examined HR+ HER2- breast cancer, and the remaining studies included patients with various breast cancer subtypes. Detailed information on the included patients is provided in **supplementary Table S3**. The ctDNA detection strategies and testing time points of each included study are summarized in **Supplementary Table S4**, whereas the patient selection criteria and data sources are presented in **Supplementary Table S5**.

### Sensitivity and specificity of landmark strategy

**Figure 2:**
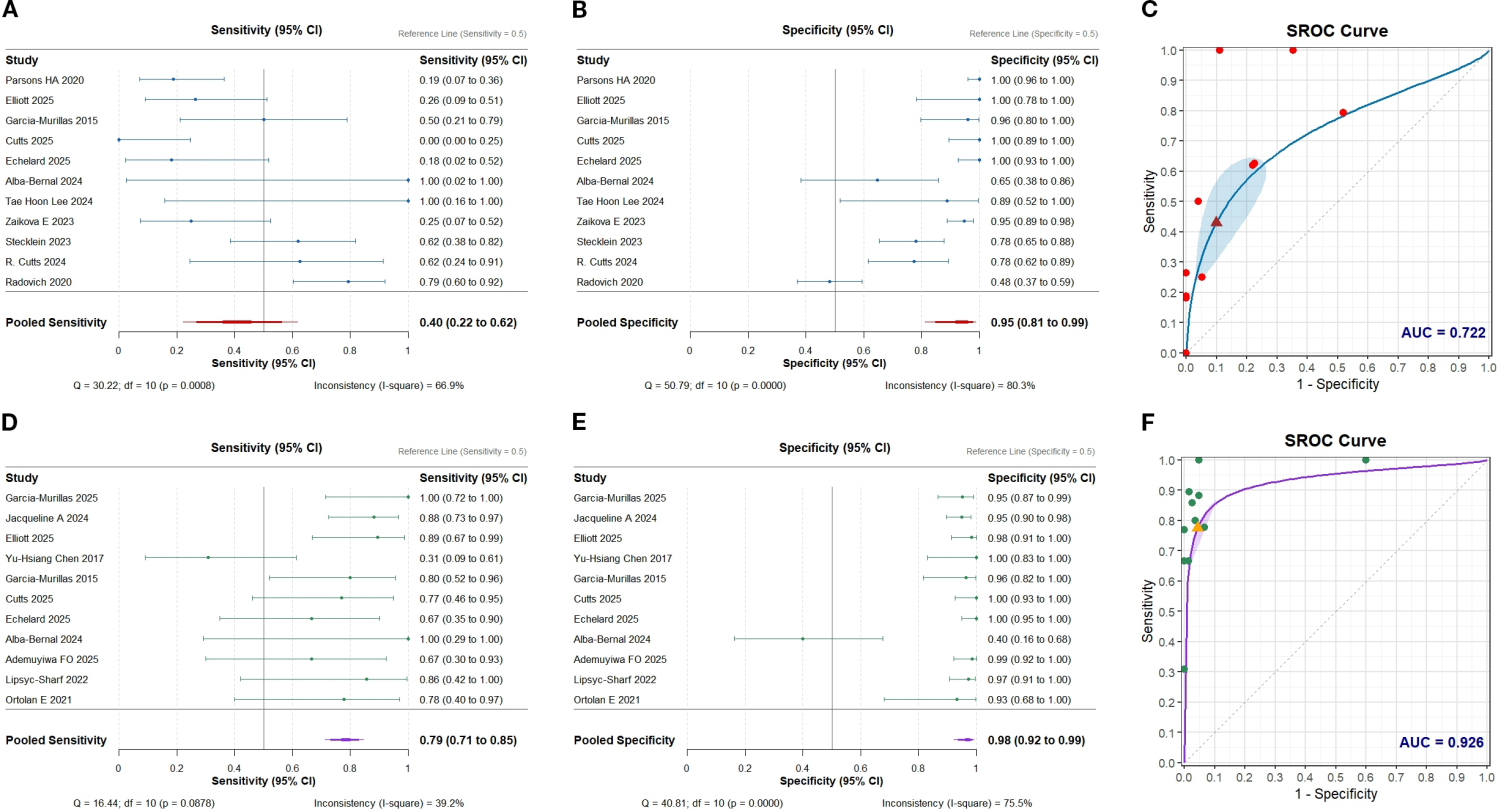
Performance of circulating tumor DNA (ctDNA) analysis methods in predicting breast cancer recurrence. Error bars represent 95% confidence intervals. **(A)** Summary of sensitivity for landmark strategies; **(B)** Summary of specificity for landmark strategies; **(C)** SROC curve for landmark strategies; **(D)** Summary of sensitivity for surveillance strategies; **(E)** Summary of specificity for surveillance strategies; **(F)** SROC curve for surveillance strategies.

In the landmark strategy, the sensitivity of ctDNA for predicting breast cancer recurrence was 0.40 (95% CI: 0.22–0.62; **Figure 2A**), while the specificity was 0.95 (95% CI: 0.81–0.99; **Figure 2B**). The area under the summary receiver operating characteristic (SROC) curve (AUC) was 0.722 (**Figure 2C**), indicating high specificity but limited sensitivity. The PLR, NLR, and DOR were 3.11 (95% CI: 1.96–4.94), 0.75 (95% CI: 0.64–0.88), and 6.03 (95% CI: 3.61–10.08), respectively.

Subgroup analysis based on molecular subtype revealed that ctDNA demonstrated the highest diagnostic performance in predicting the recurrence of TNBC, showing a sensitivity of 0.59 (95% CI: 0.49–0.69), specificity of 0.85 (95% CI: 0.70–0.94), and an AUC of 0.852. In contrast, ctDNA showed a markedly low sensitivity for HER2+ breast cancer (0.00, 95% CI: 0.00–0.60).

**Figure 3:**
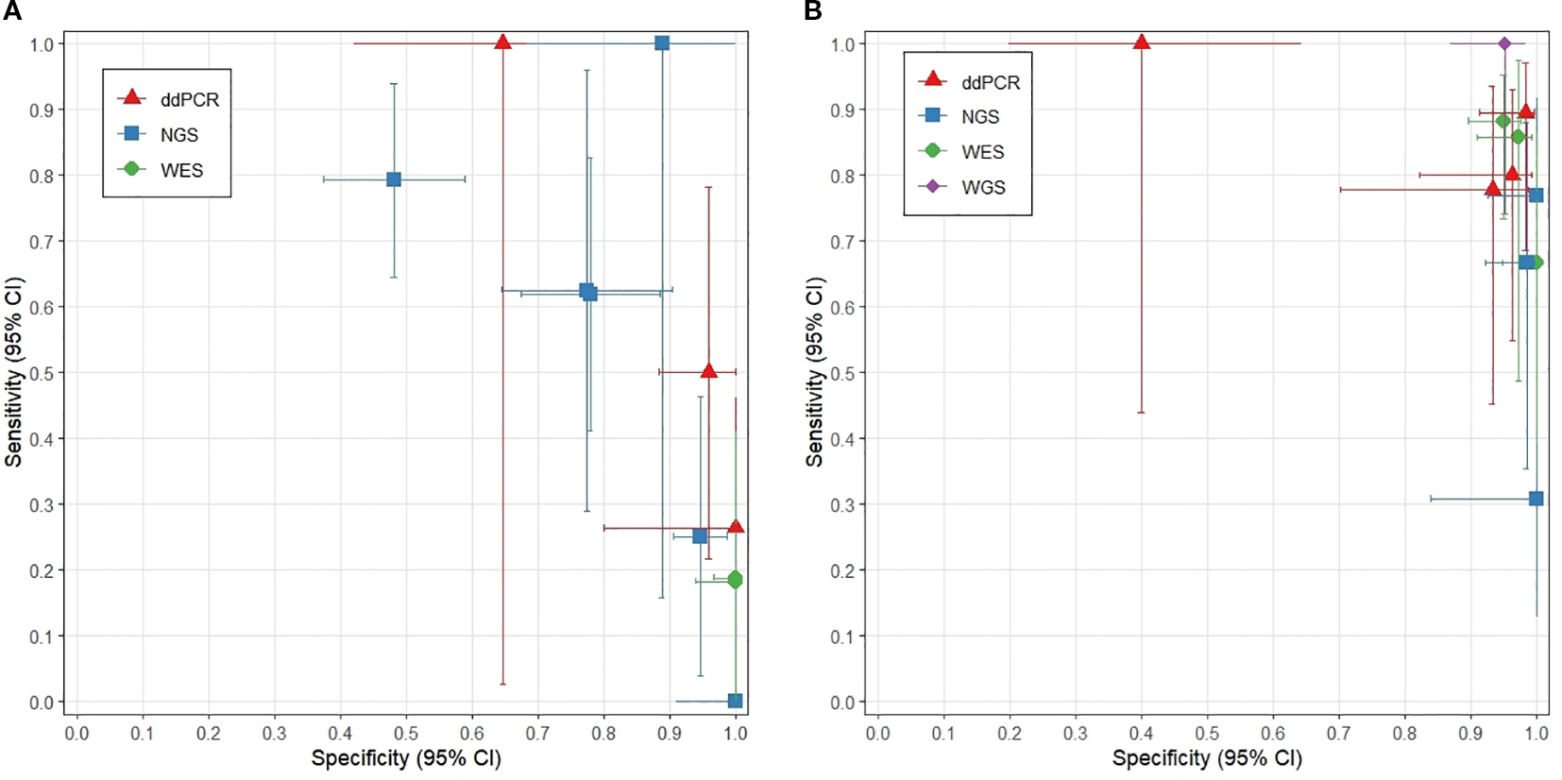
**(A)** Sensitivity and specificity analysis of landmark strategies; **(B)** Sensitivity and specificity analysis of surveillance strategies.

**Figure 4:**
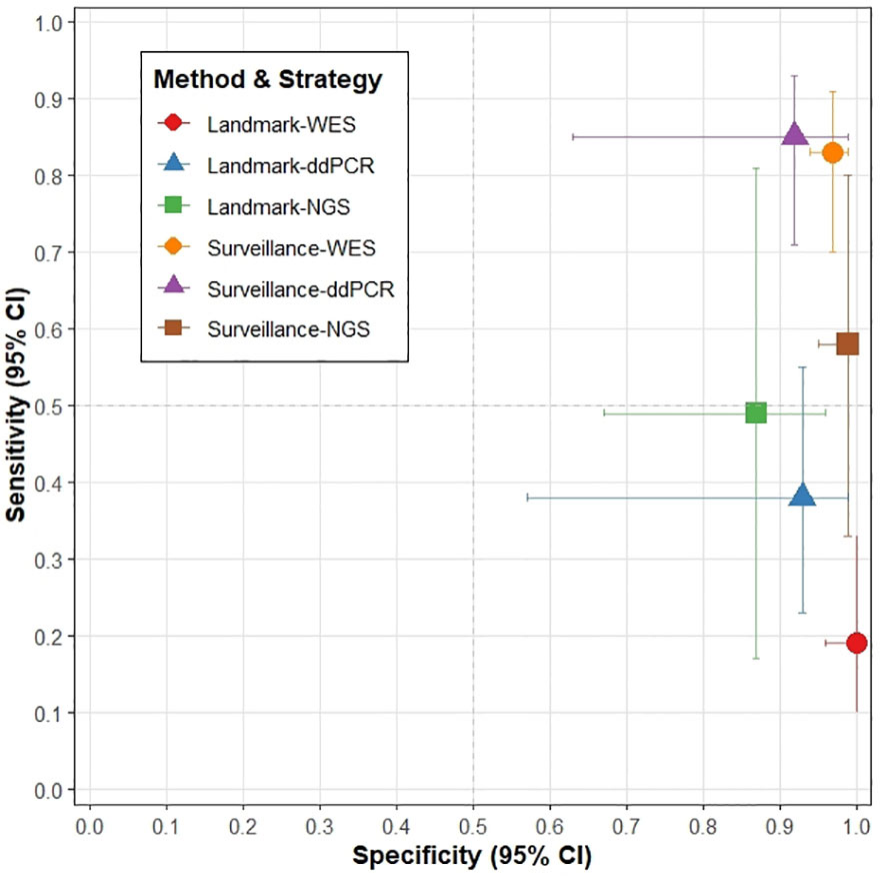
Sensitivity and specificity analysis of different detection methods across various testing strategies.

In the landmark analysis strategy, the sensitivity of all detection methods was generally low. The sensitivity was 0.49 (95% CI: 0.17–0.81) for NGS and 0.38 (95% CI: 0.23–0.55) for ddPCR. Although WES achieved perfect specificity (1.00, 95% CI: 0.97–1.00), its sensitivity was the lowest, at 0.19 (95% CI: 0.10–0.33) (**Figures 3A**, **4**).

To further evaluate the performance of each detection method across molecular subtypes, we conducted secondary subgroup analyses by stratifying the detection methods within each subtype. Because of small sample sizes in certain subgroups, limited data from individual studies, and the presence of numerous zero values, AUCs could not be calculated for some categories, resulting in pooled estimates with wide confidence intervals. Therefore, these findings should be interpreted as exploratory (**Supplementary Table S6**).

With the exception of several TNBC cases, most studies employed tumor-informed strategies. In TNBC, the tumor-uninformed strategy achieved higher sensitivity (0.62, 95% CI: 0.35–0.84), specificity (0.82, 95% CI: 0.57–0.94), and AUC (0.727) compared with the tumor-informed strategy, which showed a sensitivity of 0.48 (95% CI: 0.29–0.68), specificity of 0.72 (95% CI: 0.57–0.84), and an AUC of 0.717.

In studies in which stage III patients constituted ≥25.5% of the cohort, the sensitivity was only 0.21 (95% CI: 0.13–0.33). In contrast, in studies with <25.5% stage III patients, the sensitivity increased to 0.64 (95% CI: 0.42–0.81).

### Sensitivity and specificity of surveillance strategy

In the ctDNA surveillance strategy for predicting breast cancer recurrence, the pooled sensitivity was 0.79 (95% CI: 0.71–0.85;**Figure 2D**) and the specificity was 0.98 (95% CI: 0.92–0.99; **Figure 2E**), yielding an AUC of 0.926 (**Figure 2F**). The PLR, NLR, and DOR were 16.40 (95% CI: 6.39–42.08), 0.27 (95% CI: 0.17–0.42), and 96.05 (95% CI: 49.56–186.15), respectively.

Subgroup analysis based on molecular subtypes indicated that ctDNA performed well across all subtypes, with the best diagnostic performance observed in HR+ HER2- breast cancer (sensitivity: 0.90 [95% CI: 0.79–0.96]; specificity: 0.94 [95% CI: 0.78–0.99]).

In the subgroup analysis stratified by detection method, only one study employed WGS (9), which demonstrated nearly perfect sensitivity (1.00 [95% CI: 0.72–1.00]) and high specificity (0.95 [95% CI: 0.88–0.99]). Both WES and ddPCR also exhibited high sensitivity (0.83 [95% CI: 0.70–0.91] and 0.85 [95% CI: 0.71–0.93], respectively). In contrast, NGS showed exceptionally high specificity (0.99 [95% CI: 0.95–1.00]) but comparatively lower sensitivity (0.58 [95% CI: 0.33–0.80]) (**Figures 3B**, **4**).

In TNBC, only one study employed a tumor-agnostic strategy, achieving a sensitivity of 0.67 (95% CI: 0.30–0.93) and a specificity of 0.99 (95% CI: 0.92–1.00) (19). In contrast, studies employing tumor-informed strategies reported a pooled sensitivity of 0.71 (95% CI: 0.56–0.82), specificity of 0.96 (95% CI: 0.90–0.98), and an AUC of 0.892.

In studies in which ≥25.5% of participants had stage III breast cancer, the sensitivity reached 0.85 (95% CI: 0.74–0.91). In contrast, in studies with <25.5% stage III patients, the sensitivity was 0.84 (95% CI: 0.68–0.93), suggesting that dynamic ctDNA monitoring is particularly important for high-risk patients (**Table 1**).

**Table 1:** Results of the meta-analysis for landmark and surveillance in different groups.

Subgroup analysis		Landmark							Surveillance						
		TP	FP	FN	TN	Sensitivity	Specificity	AUC	TP	FP	FN	TN	Sensitivity	Specificity	AUC
Breast cancer type	Breast cancer	67	79	97	455	0.40 (0.22-0.62)	0.95 (0.81-0.99)	0.722	114	24	31	566	0.79 (0.71-0.85)	0.98 (0.92-0.99)	0.926
	TNBC	51	67	35	231	0.59 (0.49-0.69)	0.85 (0.70-0.94)	0.852	41	7	9	171	0.82 (0.69-0.90)	0.98 (0.87-1.00)	0.820
	HER2+	0	2	4	43	0.00 (0.00-0.60)	0.96 (0.84-0.99)	0.446	11	4	4	155	0.73 (0.47-0.90)	0.97 (0.93-0.99)	0.945
	HR+ HER2-	10	12	14	130	0.42 (0.24-0.62)	0.94 (0.72-0.99)	0.737	47	10	5	131	0.90 (0.79-0.96)	0.94 (0.78-0.99)	0.886
Detection method	WGS								11	3	0	60	1.00 (0.72-1.00)	0.95 (0.88-0.99)	NA
	WES	8	0	35	139	0.19 (0.10-0.33)	1.00 (0.97-1.00)	0.688	44	8	9	262	0.83 (0.70-0.91)	0.97 (0.94-0.99)	0.962
	ddPCR	12	7	0	50	0.38 (0.23-0.55)	0.93 (0.57-0.99)	0.724	39	12	7	108	0.85 (0.71-0.93)	0.92 (0.63-0.99)	0.818
	NGS	47	72	42	266	0.49 (0.17-0.81)	0.87 (0.67-0.96)	0.728	20	1	15	136	0.58 (0.33-0.80)	0.99 (0.95-1.00)	0.979
Detection strategy	Tumor-informed(Breast cancer)	25	16	71	253	0.26 (0.18-0.36)	0.94 (0.91-0.96)	0.693	108	23	28	498	0.79 (0.72-0.85)	0.97 (0.91-0.99)	0.925
	Tumor-informed(TNBC)	9	4	9	29	0.48 (0.29-0.68)	0.72 (0.57-0.84)	0.717	32	5	13	109	0.71 (0.56-0.82)	0.96 (0.90-0.98)	0.892
	Tumor-agnostic (TNBC)	42	63	26	194	0.62 (0.35-0.84)	0.82 (0.57-0.94)	0.727	6	1	3	68	0.67 (0.30-0.93)	0.99 (0.92-1.00)	NA
Stage	Patients at stage III > 25.5%	13	0	49	154	0.21 (0.13-0.33)	1.00 (0.98-1.00)	0.228	61	9	11	323	0.85 (0.74-0.91)	0.97 (0.95-0.99)	0.966
	Patients at stage III < 25.5%	14	19	8	57	0.64 (0.42-0.81)	0.75 (0.64-0.83)	0.741	27	14	5	148	0.84 (0.68-0.93)	0.92 (0.64-0.99)	0.853

TP true positive, FP false positive, FN false negative, TN true negative, AUC area under curve, NA, not applicable

### Heterogeneity assessment

We evaluated publication bias among the included studies using a funnel plot constructed from the DOR. The funnel plots derived from both the landmark and cumulative analyses demonstrated an approximately symmetrical distribution of study points, suggesting a low risk of publication bias (**Supplementary Figure S2**). Despite this, the 17 studies included in the analysis exhibited substantial clinical and methodological heterogeneity. The primary sources of heterogeneity were variations in study design, patient staging, molecular subtype distribution, timing of postoperative ctDNA testing, and detection methodologies. Such heterogeneity may have contributed to variability in the pooled estimates of sensitivity and specificity. Future research should focus on standardizing testing protocols to enhance the comparability and reliability of results.

## Discussion

Our study systematically evaluated the diagnostic performance of ctDNA in predicting postoperative breast cancer recurrence and, for the first time, compared the differences between landmark and surveillance analyses. Our results demonstrate that ctDNA is a highly specific biomarker associated with poor postoperative outcomes in breast cancer patients (25). Moreover, its predictive accuracy largely depends on the detection strategy used.

The relatively low sensitivity of the landmark analysis underscores the intrinsic challenge of detecting ctDNA at a single time point. This may be attributed to the low tumor burden present shortly after surgery or adjuvant therapy, which leads to a low concentration of ctDNA that remains difficult to detect. In contrast, the surveillance analysis demonstrated higher sensitivity, indicating that tumor recurrence is a dynamic and evolving process. These findings suggest that longitudinal monitoring of ctDNA provides significant value for improving the detection of postoperative recurrence in breast cancer.

Analysis of molecular subgroups revealed that the intrinsic biological characteristics of tumors significantly influence ctDNA detection rates. TNBC exhibited high detection performance under both strategies, likely owing to its inherently elevated tumor mutational burden and active DNA release properties (26). In contrast, HER2+ breast cancer displayed low sensitivity in the landmark analysis but showed a marked improvement during surveillance. This improvement may result from the introduction of targeted therapy, which efficiently eliminates most tumor cells in HER2+ breast cancer soon after treatment, thereby requiring more frequent monitoring to detect residual ctDNA signals. Furthermore, the high sensitivity and specificity observed in HR+ HER2- breast cancer during surveillance underscore the unique potential of ctDNA for predicting postoperative recurrence in this subtype.

Regarding detection technologies, all methods showed low sensitivity in the landmark analysis, emphasizing the technical challenges of detecting low levels of ctDNA at a single time point. In contrast, the diagnostic performance of these methods improved substantially in the surveillance analysis. WGS achieved exceptionally high sensitivity and specificity in the surveillance analysis; however, its high cost may currently restrict widespread clinical adoption (27). Notably, NGS showed lower sensitivity than ddPCR and WES in the surveillance analysis, likely due to its reliance on fixed gene panels. If the recurrent tumor clones harbor mutations outside the coverage of the panel, false negatives may occur. This finding underscores the considerable challenge posed by tumor clonal evolution in ctDNA monitoring and suggests that future research should prioritize developing individualized, tumor-informed NGS detection strategies. Our data revealed that tumor-uninformed strategies demonstrated markedly higher sensitivity than tumor-informed strategies in the landmark analysis. This difference may arise because, at a single postoperative time point with an extremely low tumor burden, tumor-uninformed strategies employ ultra-deep sequencing, thereby improving genomic coverage and mutation detection sensitivity. In contrast, during surveillance, tumor-informed strategies achieved more stable and superior sensitivity by continuously tracking individualized mutations. These findings suggest that tumor-uninformed strategies are better suited for single-point risk screening, whereas tumor-informed strategies are preferable for long-term dynamic monitoring. Future studies should explore phased combination strategies to balance initial screening efficiency and long-term monitoring accuracy.

Similar findings have been reported for ctDNA monitoring in other cancer types. In colorectal cancer, dynamic monitoring strategies significantly enhance the sensitivity of recurrence prediction compared with single time-point testing, and tumor-informed approaches demonstrate superior performance in long-term surveillance (28). Likewise, in lung cancer, postoperative ctDNA monitoring has been shown to identify recurrence earlier than conventional imaging, with longitudinal monitoring strategies exhibiting greater sensitivity than single time point detection (29). Collectively, these findings indicate that dynamic ctDNA monitoring has broad applicability for recurrence surveillance across multiple malignancies, further supporting its potential value in the postoperative management of breast cancer.

We found that in studies where more than 25.5% of patients were at stage III, the sensitivity of the landmark analysis was markedly low. This suggests that high-risk patients may have eliminated most drug-sensitive and rapidly proliferating tumor cells with high ctDNA release through neoadjuvant therapy. In contrast, the sensitivity of the surveillance analysis increased substantially, underscoring the indispensable role of dynamic monitoring in postoperative ctDNA detection among high-risk patients. Conversely, in studies where fewer than 25.5% of patients were at stage III, the baseline sensitivity of the landmark analysis was relatively high. This may be because intermediate- and low-risk patients underwent direct surgery without neoadjuvant therapy, thus retaining the active proliferative characteristics of the primary tumor and making ctDNA easier to detect. The surveillance analysis further improved both sensitivity and specificity. High-risk patients should rely on dynamic monitoring to minimize the risk of missed diagnoses, whereas intermediate- and low-risk patients may benefit from a combined monitoring model that integrates landmark and surveillance analyses. Such an approach could inform the development of individualized adjuvant therapy and follow-up strategies.

In summary, this study provides a comprehensive evaluation of ctDNA performance in predicting postoperative breast cancer recurrence. Although ctDNA showed high specificity in predicting breast cancer recurrence, its sensitivity remained suboptimal, especially in landmark analyses. In the future, detection efficiency could be enhanced through several approaches, including optimization of ctDNA extraction, reduction of background noise, and tracking of multi-locus gene mutations (6, 27, 30). Beyond technological improvements, integrating ctDNA with other liquid biopsy biomarkers, such as circulating tumor cells (CTCs) and exosomes, may further improve overall sensitivity and clinical utility in recurrence risk prediction (24, 31). Future research should prioritize standardizing detection methods across molecular subtypes, optimizing testing strategies, and implementing multidimensional evaluations to advance precision and personalized postoperative management of breast cancer through ctDNA monitoring.

### Limitations

First, the total sample size across the 17 included studies was limited, and several subgroups had small cohorts, thereby reducing the statistical power and robustness of the conclusions. Second, due to the high heterogeneity of breast cancer and the lack of detailed individual-level data in most studies, we were unable to fully evaluate the potential effects of driver gene status and specific treatment regimens on the outcomes, despite performing subgroup analyses by molecular subtype, detection method, stage, and strategy. These factors may influence the accuracy of postoperative recurrence risk assessment in breast cancer.

## Conclusion

ctDNA has been identified as a promising biomarker for predicting postoperative recurrence in patients with breast cancer. It exhibited excellent specificity in both landmark and surveillance analyses. To realize its clinical translational potential, more sensitive and cost-effective detection technologies should be developed. Optimization of detection strategies—such as designing subtype-specific detection panels—may enable ctDNA monitoring to provide crucial evidence for precise risk stratification and personalized adjuvant treatment decisions in breast cancer.

## Data Availability

The original contributions presented in the study are included in the article/**Supplementary Material**. Further inquiries can be directed to the corresponding author.
