# Supplementary material for: Accuracy of ctDNA-based minimal residual disease detection in predicting postoperative recurrence of breast cancer: a meta-analysis
Source: Front Oncol. 2026 Feb 3;16:1735752. doi: 10.3389/fonc.2026.1735752 (PMC12909176; doi:10.3389/fonc.2026.1735752)
Supplement: Supplementary file 4 [file DataSheet1.pdf]

1 Supplementary Figures and Tables

1.1. Supplementary Figures

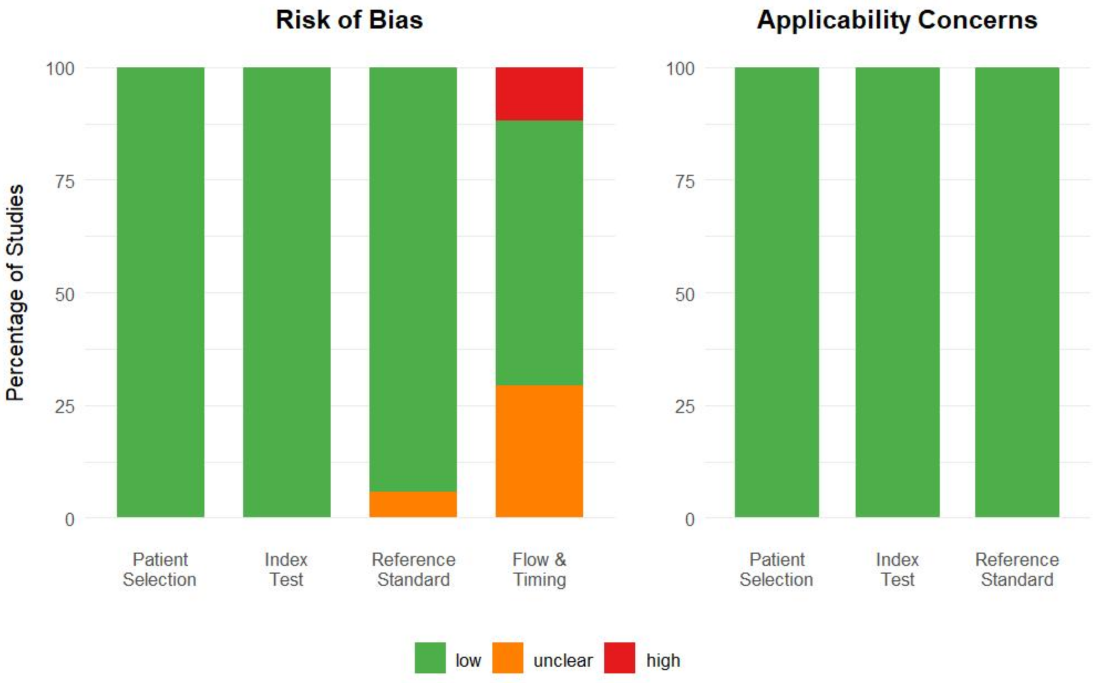

(A)

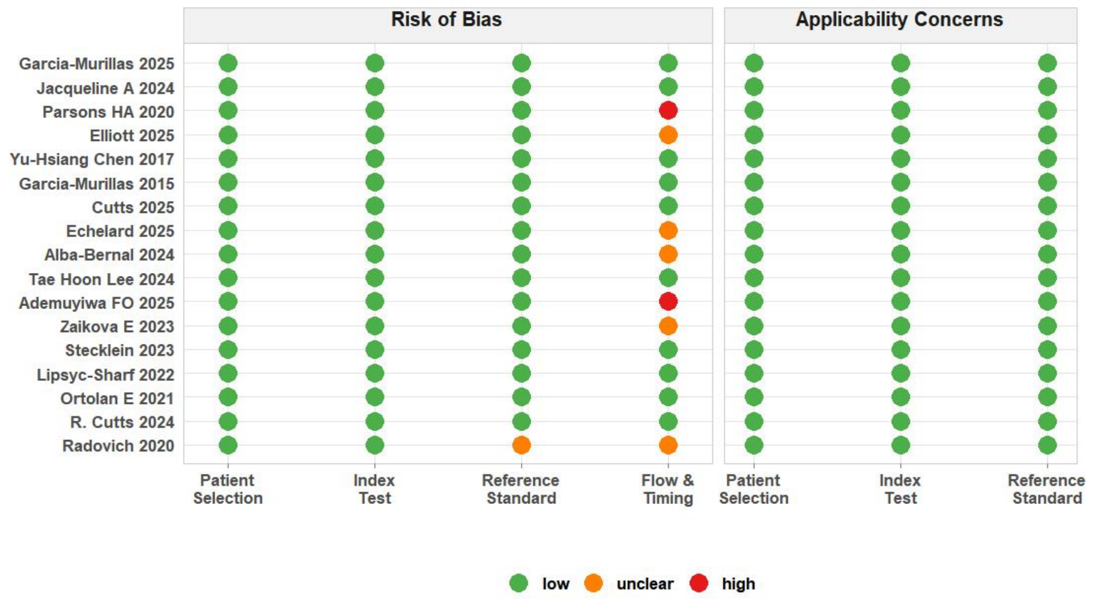

(B)

Supplementary Figure S1. (A) Methodological quality assessment graph. The evaluated domains included patient selection, index test, reference standard, and follow-up duration. All four domains contributed to the evaluation of risk of bias, whereas the first three were additionally used to determine applicability concerns. **(B)** Methodological quality summary. A detailed overview of the risk of bias and applicability concerns for each included study is provided.

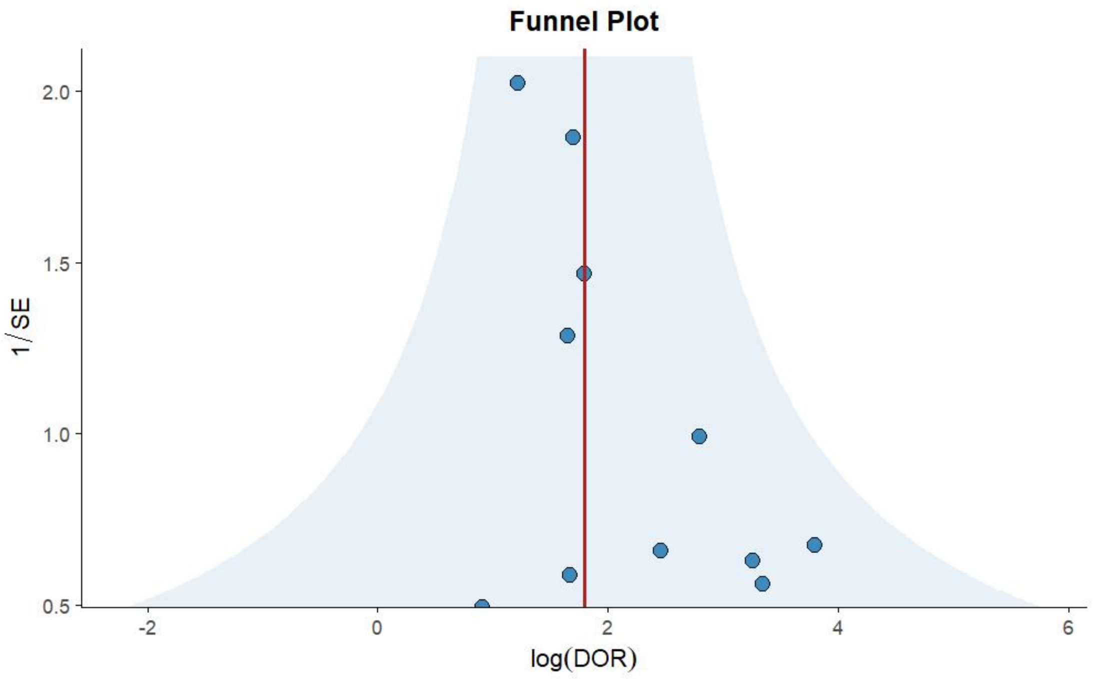

(A)

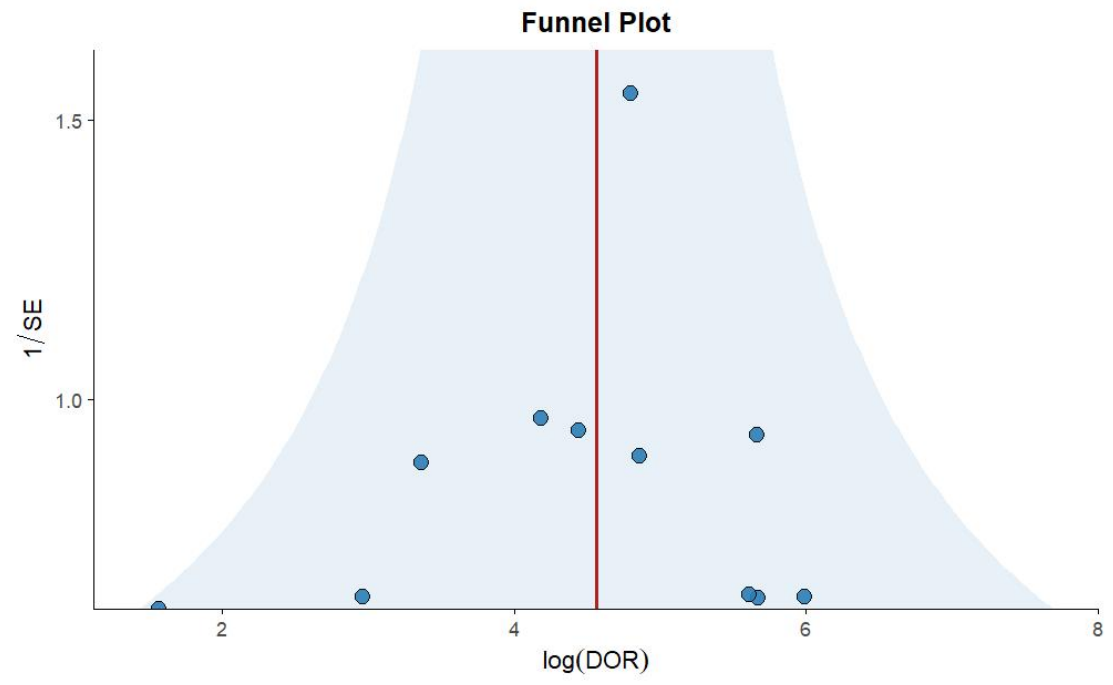

(B)

Supplementary Figure S2. Funnel plots based on diagnostic odds ratio (DOR). The blue area represents the funnel, and each circle denotes an individual study. **(A)** Funnel plot for the landmark strategy. **(B)** Funnel plot for the surveillance strategy.

1.2. Supplementary Table

Supplementary Table S1. search strategy.

Cochrane		
ID	Search	Result
#1	MeSH descriptor: [Breast Neoplasms] explode all trees	20565
#2	(‘ bilateral breast neoplasm' OR 'bilateral breast tum*' OR 'breast cancer' OR 'breast carcinoma*' OR 'breast gland tum*' OR 'breast malignant neoplasm*' OR 'breast malignant tumor*' OR 'breast mass' OR 'breast neoplasia' OR 'breast neoplasm*' OR 'breast tum*' OR 'cancer of breast' OR 'cancer of the breast' OR 'female breast neoplasm' OR 'female breast tum*' OR 'human mammary carcinoma*' OR 'human mammary neoplasm*' OR 'male breast neoplasm' OR 'male breast tum*' OR 'malignant neoplasm of breast' OR 'malignant tumor of breast' OR 'mamma tum*' OR 'mammary cancer*' OR 'mammary gland neoplasia' OR 'mammary gland neoplasm' OR 'mammary gland tum*' OR 'mammary neoplasia' OR 'mammary neoplasm*' OR 'mammary tumor' OR 'mammary tumor cell' OR 'mammary tumorigenesis' OR 'mammary tumour' OR 'mammary tumour cell' OR 'mass in the breast' OR 'masses in the breast' OR 'neoplasia of the breast' OR 'neoplasm of the breast' OR 'neoplasm of the mammary gland' OR 'neoplastic breast' OR 'neoplastic mammary' OR 'neoplastic mammary gland' OR 'tumor of the breast' OR 'tumor of the female breast' OR 'tumor of the male breast' OR 'tumor of the mammary gland' OR 'tumorigenesis of the breast' OR 'tumorigenesis of the mammary gland' OR 'tumour of the male breast' OR 'unilateral breast neoplasm*' OR 'unilateral breast tumor'):ti,kw,ab	51509
#3	MeSH descriptor: [Circulating Tumor DNA] explode all trees	109
#4	MeSH descriptor: [Cell-Free Nucleic Acids] explode all trees	235
#5	MeSH descriptor: [Liquid Biopsy] explode all trees	27
#6	MeSH descriptor: [DNA, Neoplasm] explode all trees	382
#7	(' cell free deoxyribonucleic acid' OR 'cell free dna' OR 'cell free nucleic acid*' OR 'cell free ribonucleic acid' OR 'cell free rna' OR 'cfdna' OR 'cfrna' OR 'circulating dna' OR 'circulating nucleic acid*' OR 'circulating rna' OR 'cirdna' OR 'cirrna' OR 'ctDNA' OR 'fluid biopsy' OR 'liquid biops*' OR 'Neoplasm DNA'):ti,kw,ab	7879
#8	MeSH descriptor: [Neoplasm, Residual] explode all trees	602
#9	(‘ minimal residual disease*' OR 'minimum residual disease' OR 'MRD' OR 'residual cancer*' OR 'residual disease' OR 'residual minimal disease*' OR 'residual neoplasm*' OR 'residual tumor*' OR 'residual tumour*'):ti,kw,ab	12862
#10	#1 OR #2	51509
#11	#3 OR #4 OR #5 OR #6 OR #7	7880
#12	#8 OR #9	12862
#13	#10 AND #11 AND #12 with Cochrane Library publication date Between Jan 2000 and Apr 2025	88

Embase		
ID	Search	Results
#14	#10 AND #11 AND #12 AND [2000-2025]/py	450
#13	#10 AND #11 AND #12	450
#12	#8 OR #9	80393
#11	#3 OR #4 OR #5 OR #6 OR #7	52212
#10	#1 OR #2	821773
#9	'minimal residual disease*':ti,ab,kw OR 'minimum residual disease':ti,ab,kw OR 'mrd':ti,ab,kw OR 'residual cancer*':ti,ab,kw OR 'residual disease':ti,ab,kw OR 'residual minimal disease*':ti,ab,kw OR 'residual neoplasm*':ti,ab,kw OR 'residual tumor*':ti,ab,kw OR 'residual tumour*':ti,ab,kw	70658
#8	'minimal residual disease'/exp	46905
#7	'cell free deoxyribonucleic acid':ti,ab,kw OR 'cell free dna':ti,ab,kw OR 'cell free nucleic acid*':ti,ab,kw OR 'cell free ribonucleic acid':ti,ab,kw OR 'cell free rna':ti,ab,kw OR 'cfdna':ti,ab,kw OR 'cfrna':ti,ab,kw OR 'circulating dna':ti,ab,kw OR 'circulating nucleic acid*':ti,ab,kw OR 'circulating rna':ti,ab,kw OR 'cirdna':ti,ab,kw OR 'cirrna':ti,ab,kw OR 'ctdna':ti,ab,kw OR 'fluid biopsy':ti,ab,kw OR 'liquid biops*':ti,ab,kw OR 'neoplasm dna':ti,ab,kw	42713
#6	'circulating free dna'/exp	8007
#5	'liquid biopsy'/exp	15571
#4	'cell free nucleic acid'/exp	24613
#3	'circulating tumor dna'/exp	13117
#2	'bilateral breast neoplasm':ti,ab,kw OR 'bilateral breast tum*':ti,ab,kw OR 'breast cancer':ti,ab,kw OR 'breast carcinoma*':ti,ab,kw OR 'breast gland tum*':ti,ab,kw OR 'breast malignant neoplasm*':ti,ab,kw OR 'breast malignant tumor*':ti,ab,kw OR 'breast mass':ti,ab,kw OR 'breast neoplasia':ti,ab,kw OR 'breast neoplasm*':ti,ab,kw OR 'breast tum*':ti,ab,kw OR 'cancer of breast':ti,ab,kw OR 'cancer of the breast':ti,ab,kw OR 'female breast neoplasm':ti,ab,kw OR 'female breast tum*':ti,ab,kw OR 'human mammary carcinoma*':ti,ab,kw OR 'human mammary neoplasm*':ti,ab,kw OR 'male breast neoplasm':ti,ab,kw OR 'male breast tum*':ti,ab,kw OR 'malignant neoplasm of breast':ti,ab,kw OR 'malignant tumor of breast':ti,ab,kw OR 'mamma tum*':ti,ab,kw OR 'mammary cancer*':ti,ab,kw OR 'mammary gland neoplasia':ti,ab,kw OR 'mammary gland neoplasm':ti,ab,kw OR 'mammary gland tum*':ti,ab,kw OR 'mammary neoplasia':ti,ab,kw OR 'mammary neoplasm*':ti,ab,kw OR 'mammary tumor':ti,ab,kw OR 'mammary tumor cell':ti,ab,kw OR 'mammary tumorigenesis':ti,ab,kw OR 'mammary tumour':ti,ab,kw OR 'mammary tumour cell':ti,ab,kw OR 'mass in the breast':ti,ab,kw OR 'masses in the breast':ti,ab,kw OR 'neoplasia of the breast':ti,ab,kw OR 'neoplasm of the breast':ti,ab,kw OR 'neoplasm of the mammary gland':ti,ab,kw OR 'neoplastic breast':ti,ab,kw OR 'neoplastic mammary':ti,ab,kw OR 'neoplastic mammary gland':ti,ab,kw OR 'tumor of the breast':ti,ab,kw OR 'tumor of the female breast':ti,ab,kw OR 'tumor of the male breast':ti,ab,kw OR 'tumor of the mammary gland':ti,ab,kw OR 'tumorigenesis of the breast':ti,ab,kw OR 'tumorigenesis of the mammary gland':ti,ab,kw OR 'tumour of the male breast':ti,ab,kw OR 'unilateral breast neoplasm*':ti,ab,kw OR 'unilateral breast tumor':ti,ab,kw	612910
#1	'breast tumor'/exp	751155

Ovid		
ID	Search	Result
1	Breast Neoplasms/	352805
2	(bilateral breast neoplasm or bilateral breast tum* or breast cancer or breast carcinoma* or breast gland tum* or breast malignant neoplasm* or breast malignant tumor* or breast mass or breast neoplasia or breast neoplasm* or breast tum* or cancer of breast or cancer of the breast or female breast neoplasm or female breast tum* or human mammary carcinoma* or human mammary neoplasm* or male breast neoplasm or male breast tum* or malignant neoplasm of breast or malignant tumor of breast or mamma tum* or mammary cancer* or mammary gland neoplasia or mammary gland neoplasm or mammary gland tum* or mammary neoplasia or mammary neoplasm* or mammary tumor or mammary tumor cell or mammary tumorigenesis or mammary tumour or mammary tumour cell or mass in the breast or masses in the breast or neoplasia of the breast or neoplasm of the breast or neoplasm of the mammary gland or neoplastic breast or neoplastic mammary or neoplastic	424007

	mammary gland or tumor of the breast or tumor of the female breast or tumor of the male breast or tumor of the mammary gland or tumorigenesis of the breast or tumorigenesis of the mammary gland or tumour of the male breast or unilateral breast neoplasm* or unilateral breast tumor).ab,ti,kw.	
3	Circulating Tumor DNA/	3453
4	Cell-Free Nucleic Acids/	4407
5	Liquid Biopsy/	3517
6	DNA, Neoplasm/	44953
7	(cell free deoxyribonucleic acid or cell free dna or cell free nucleic acid* or cell free ribonucleic acid or cell free rna or cfdna or cfrna or circulating dna or circulating nucleic acid* or circulating rna or cirdna or cirrna or ctDNA or fluid biopsy or liquid biops* or Neoplasm DNA).ab,ti,kw.	22919
8	Neoplasm, Residual/	13533
9	(minimal residual disease* or minimum residual disease or MRD or residual cancer* or residual disease or residual minimal disease* or residual neoplasm* or residual tumor* or residual tumour*).ab,ti,kw.	36953
10	1 or 2	503095
11	3 or 4 or 5 or 6 or 7	68058
12	8 or 9	41860
13	10 and 11 and 12	150
14	limit 13 to yr="2000 - 2025"	138

Pubmed			
ID	Search	Filters	Results
8	(#1 OR #2) AND (#3 OR #4) AND (#5 OR #6)	from 2000 - 2025	158
7	(#1 OR #2) AND (#3 OR #4) AND (#5 OR #6)		171
6	"minimal residual disease*"OR"minimum residual disease"OR"MRD"OR"residual cancer*"OR"residual disease"OR"residual minimal disease*"OR"residual neoplasm*"OR"residual tumor*"OR"residual tumour*"[Title/Abstract]		42,668
5	Neoplasm, Residual[MeSH Terms]		13,522
4	"cell free deoxyribonucleic acid"OR"cell free dna"OR"cell free nucleic acid*"OR"cell free ribonucleic acid"OR"cell free rna"OR"cfdna"OR"cfrna"OR"circulating dna"OR"circulating nucleic acid*"OR"circulating rna"OR"cirdna"OR"cirrna"OR"ctDNA"OR"fluid biopsy"OR"liquid biops*"OR"Neoplasm DNA"[Title/Abstract]		26,246
3	"Circulating Tumor DNA"OR"Cell-Free Nucleic Acids"OR"Liquid Biopsy"OR"DNA, Neoplasm"[MeSH Terms]		62,747
2	"bilateral breast neoplasm"OR"bilateral breast tum*"OR"breast cancer"OR"breast carcinoma*"OR"breast gland tum*"OR"breast malignant neoplasm*"OR"breast malignant tumor*"OR"breast mass"OR"breast neoplasia"OR"breast neoplasm*"OR"breast tum*"OR"cancer of breast"OR"cancer of the breast"OR"female breast neoplasm"OR"female breast tum*"OR"human mammary carcinoma*"OR"human mammary neoplasm*"OR"male breast neoplasm"OR"male breast tum*"OR"malignant neoplasm of breast"OR"malignant tumor of breast"OR"mamma tum*"OR"mammary cancer*"OR"mammary gland neoplasia"OR"mammary gland neoplasm"OR"mammary gland tum*"OR"mammary neoplasia"OR"mammary neoplasm*"OR"mammary tumor"OR"mammary tumor cell"OR"mammary tumorigenesis"OR"mammary tumour"OR"mammary tumour cell"OR"mass in the breast"OR"masses in the breast"OR"neoplasia of the breast"OR"neoplasm of the breast"OR"neoplasm of the mammary gland"OR"neoplastic breast"OR"neoplastic mammary"OR"neoplastic mammary gland"OR"tumor of the breast"OR"tumor of the female breast"OR"tumor of the male breast"OR"tumor of the mammary gland"OR"tumorigenesis of the breast"OR"tumorigenesis of the mammary gland"OR"tumour of		545,494

	the male breast"OR"unilateral breast neoplasm*"OR"unilateral breast tumor"[Title/Abstract]	
1	"Breast Neoplasms"[Mesh]	366,840

Supplementary Table S4. ctDNA detection time points of each included study.

Study	Surgery	1 week	2 weeks	3 weeks	4 weeks	1 month	6 weeks	8 weeks	60 days	10 weeks	3 months	14 weeks	6 months	7 months	12 months	18 months	24 months	...
Garcia-Murillas 2025		14 days		every 3 months during the first year, and every 6 months thereafter														
Jacqueline A 2024											every 6 months							
Parsons HA 2020															1 year			
Elliott 2025		within 60 days								≥60 days with a planned frequency of every 6 to 12 months								
Yu-Hsiang Chen 2017				Cycles 1 and 2 of the combination phase,and during weeks 1 and 5 of the maintenance phase														
Garcia-Murillas 2015			2-4 weeks			Every 6 months during follow-up												
Cutts 2025		≤8 weeks							Every 3 months during the first 2 years,then every 6 months									
Echelard 2025		within 60 days								≥60 days after surgery with a planned frequency of every 6 to 12 months								
Alba-Bernal 2024						1 month	every 6 months											
Tae Hoon Lee 2024		before radiotherapy																
Ademuyiwa FO 2025		21 days			every 6 months for 5 years													
Zaikova E 2023		within 7 months after curative treatment																
Stecklein 2023		Within 1-6 months after curative treatment																every 6-12 mont
Lipsyc-Sharf 2022																		
Ortolan E 2021											every 6 months							
R. Cutts 2024		1-14 weeks before adjuvant therapy																
Radovich 2020		at time before adjuvant chemotherapy																

Landmark

Surveillance

Excluded

Supplementary Table S6. Results of Meta-Analysis for Each Detection Method Across Different Molecular Subtypes

**Subgroup analysis**		**Landmark**							**surveillance**						
		**TP**	**FP**	**FN**	**TN**	**Sensitivity**	**Specificity**	**AUC**	**TP**	**FP**	**FN**	**TN**	**Sensitivity**	**Specificity**	**AUC**
Detection method	WGS（TNBC）								3	1	0	17	1.00 (0.29–1.00)	0.94 (0.73–1.00)	NA
	WES（TNBC）	8	0	35	139	0.19 (0.10-0.33)	1.00 (0.97-1.00)	0.688	44	8	9	262	0.83 (0.70-0.91)	0.97 (0.94-0.99)	0.962
	ddPCR（TNBC）	12	7	20	50	0.38 (0.23-0.55)	0.93 (0.57-0.99)	0.724	39	12	7	108	0.85 (0.71-0.93)	0.92 (0.63-0.99)	0.818
	NGS（TNBC）	47	72	42	266	0.49 (0.17-0.81)	0.87 (0.67-0.96)	0.728	20	1	15	136	0.58 (0.33-0.80)	0.99 (0.95-1.00)	0.979
	WGS（HER2+）								3	1	0	17	1.00 (0.29–1.00)	0.94 (0.73–1.00)	NA
	WES（HER2+）	0	0	3	8	0.00 (0.00–0.71)	1.00 (0.63–1.00)	NA	8	0	2	35	0.80 (0.46–0.95)	1.00 (0.99–1.00)	0.974
	ddPCR（HER2+）	5	3	1	8	0.83 (0.37–0.98)	0.73 (0.41–0.91)	NA	14	4	1	25	0.93 (0.65–0.99)	0.86 (0.69–0.95)	0.847
	NGS（HER2+）	46	64	31	215	0.57 (0.29–0.81)	0.85 (0.65–0.95)	0.728	13	1	13	100	0.50 (0.32–0.68)	0.99 (0.93–1.00)	0.975
	WGS（HR+HER2-）								5	2	0	13	1.00 (0.48–1.00)	0.87 (0.59–0.98)	NA
	WES（HR+ HER2-）	8	2	6	90	0.59 (0.17–0.91)	0.98 (0.92–0.99)	0.968	25	3	5	88	0.83 (0.63–0.93)	0.97 (0.90–0.99)	0.966
	ddPCR（HR+ HER2-）	1	4	0	6	1.00 (0.03–1.00)	0.60 (0.26–0.88)	NA	11	5	0	15	1.00 (0.72–1.00)	0.76 (0.36–0.95)	0.912
	NGS（HR+ HER2-）	1	6	8	34	0.11 (0.02–0.50)	0.85 (0.70–0.93)	0.513	6	0	0	15	1.00 (0.54–1.00)	1.00 (0.78–1.00)	NA

Abbreviations: TP true positive, FP false positive, FN false negative, TN true negative, AUC area under curve, NA, not applicable
